# Mortality and Morbidity Among Persons Deprived of Liberty During the COVID-19 Pandemic in Brazil

**DOI:** 10.1089/heq.2021.0057

**Published:** 2021-08-19

**Authors:** Matheus Lins-Rocha, Liliane Lins-Kusterer, José Menezes, Ailton Melo

**Affiliations:** ^1^ Postgraduate Program in Medicine and Health, Faculty of Medicine, Federal University of Bahia, Salvador, Brazil.; ^2^ Postgraduate Program in Law, Governance and Public Policy, Salvador University—UNIFACS, Salvador, Brazil.

Dear Editor:

In Brazil, persons deprived of liberty (PDL) have a high risk of COVID-19 infection due to the overcrowded and deficiently ventilated cells, restricted access to basic sanitation, and a lack of health units.^1^ Brazilian health services in prisons should be distributed according to the prison population and organized following the national primary care model, achieving the universality of the Unified Health System and guaranteeing the constitutional right to health for prisoners.^2^ Brazilian prisons present considerable challenges for the implementation of preventive health policies. A recent ecological study evaluated the five Brazilian macro-regions, including the Federal District. Results showed 14 spatial risk clusters for COVID-19 among PDL, and the highest-risk cluster was in the Federal District.^1^

The health of PDL is not an object of great concern by society. The provision of specialized health services has declined during the COVID-19 pandemic in Brazil. In this context, a similar or even worse situation is expected to occur concerning the health of Brazilian prisoners. We compared general morbidity and mortality of prisoners in the State of Bahia, Brazil, in the second half of 2019 and the first half of 2020.

A retrospective study analyzed data from the Brazilian Penitentiary statistical information system (INFOPEN),^3^ for the State of Bahia, Brazil, concerning periods before (second half of 2019) and at the beginning of (first half of 2020) the COVID-19 pandemic. Prevalence of specific diseases and mortality rates for the two semesters were compared by using the OpenEpi tool (www.OpenEpi.com).^4^

Comparing the two semesters, the prevalence of other causes of illness increased substantially among men (13.1 times) and women (5.98 times). A decrease (29%) in the prevalence of tuberculosis among men was also observed. Mortality by criminal causes and suicide increased 7.92 and 3.17 times among men, comparing semesters before and during the COVID-19 pandemic. Women accounted for 2.8% and 3.3% of the prisoners' total populations, in the first half of 2019 and 2020, respectively. There was only one case of death in the first half of 2020 (Table 1).

**Table 1.** Prevalence (per 10,000) of Diseases and Mortality (per 100,000) per Cause in Persons Deprived of Liberty According to Sex and Semester, State of Bahia, Brazil

Disease/sex	2019 (second half)		2020 (first half)		PR (2020.1/2019.2)	95% CI
	***n***	Prevalence	***n***	Prevalence		
Male (*n*=14,687)			Male (*n*=13,911)			
Hepatitis	59	40.2	68	48.9	1.21	0.85–1.72
HIV	155	105.5	145	104.2	0.98	0.78–1.23
Syphilis	422	287.3	374	268.9	0.93	0.81–1.07
Tuberculosis	169	115.1	115	82.7	0.71	0.57–0.91
Others	9	6.1	112	80.5	13.1	6.67–25.90
Female (*n*=421)			Female (*n*=469)			
Hepatitis	2	47.5	1	21.3	0.45	0.04–4.93
HIV	10	237.5	10	213.2	0.90	0.38–2.13
Syphilis	18	427.5	25	533.0	1.25	0.69–2.25
Tuberculosis	2	47.5	0	0.0	—	—
Others	3	71.3	20	426.4	5.98	1.79–20.0

Cause	2019 (second half) (***n***=14,687)		2020 (first half) (***n***=13,911)		MRR (2020.1/2019.2)	95% CI
	***n***	Mortality	***n***	Mortality		
Male						
Health causes	8	54.5	14	100.6	1.85	0.77–4.40
Suicide	2	13.6	6	43.1	3.17	0.64–15.70
Criminal	4	27.2	30	215.7	7.92	2.80–22.47
Unknown	4	27.2	3	21.6	0.79	0.18–3.54
Total	18	122.6	53	381.0	3.11	1.82–5.30

Source: Sistema de Informações do Departamento Penitenciário Nacional, 2019.

MRR, mortality rate ratio; PR, prevalence ratio.

.

A report issued by the Catholic Church in Brazil entitled *The Pandemic of Torture in Prison*, published by the Prison Ministry Office of the Bishops' Conference of Brazil, registered 90 complaints of ill treatment. The complaints included physical abuse, humiliating treatments, deprivations, and violations of the constitutional right to health care in PDL.^5,6^ Most of the complaints were ignored by judiciary authorities. Of the 90 reported complaints, only 8 were followed by an investigation.^6^

The advent of the COVID-19 pandemic was associated with changes in disease prevalence and mortality among PDL in the State of Bahia penitentiary system. We emphasize the increase of violent deaths, reflecting the stressful situation of the inmates. Public Policies for PDL need to be effective in reducing the number of crimes or enabling the reintegration of prisoners into society. They should also include health accessibility, and guarantee constitutional humans rights, preventing violence and torture.

## Abbreviations Used

INFOPEN: Brazilian Penitentiary statistical information system
PDL: persons deprived of liberty
